# The outcomes of a methodology for developing prospective pharmacists' English lexical competence

**DOI:** 10.25122/jml-2022-0115

**Published:** 2022-11

**Authors:** Tomashevska Agnessa Yuriivna, Rohovyi Yurii Yevgenivich, Michael Ivanovich Sheremet

**Affiliations:** 1 Department of Foreign Languages, Bukovinian State Medical University, Chernivtsi, Ukraine; 2 Department of Pathological Physiology, Bukovinian State Medical University, Chernivtsi, Ukraine; 3 Surgical department No. 1, Bukovinian State Medical University, Chernivtsi, Ukraine

**Keywords:** prospective pharmacists, lexical competence, reading, speaking

## Abstract

This study assessed the effectiveness of a methodology aimed at developing prospective pharmacists' professionally oriented English lexical competence in reading and speaking during individual study. The methodology considered students' interests and experience, forming lexical skills in reading, speaking, listening, and writing, integration with professional subjects, learning metacognitive, cognitive, and compensatory strategies, enhancing students' autonomy through purposeful forming their reflexive abilities, developing professionally oriented English lexical competence in reading and speaking within three stages, using the suggested system of exercises that consists of corresponding groups (correlating with the stages) and subgroups. The paper also substantiates the criteria for assessing the level of prospective pharmacists in mastering professionally oriented English lexical competence in reading and speaking: the accuracy of understanding, the correctness of guessing a lexical unit's meaning, the adequacy of lexical unit's usage, and lexical concentration. In addition, the paper describes the results of the experiment and validates the data obtained with the help of the multifunctional statistic criterion ∏* – of Fishers angular transformation.

## INTRODUCTION

Considering the increasing integration of Ukraine into the world, particular emphasis should be placed on acquiring professionally oriented English lexical competence among medical students. This would enhance their access to valuable professional information and modern medical developments and facilitate communication with foreign colleagues and English-speaking patients, which is currently essential [1].

Lexical competence is an indispensable element of professionally oriented English communication. It ensures the relevant understanding of lexical units in speech, as well as their usage in the process of communication [2]. English lexical competence in reading and speaking is interpreted as a specialist's ability (based on obtained lexical knowledge, acquired lexical skills, and lexical consciousness) to recognize and perceive lexical units, understand them at the level of the word, word combination, sentence, and text [3, 4]. Moreover, any specialist should be able to adequately express their thoughts in professional communication and ensure the self-improvement of their lexical skills [5]. The above necessity is closely associated with the specifics of prospective pharmacists' professional activities. The latter requires the knowledge of certain terms, their correct understanding, and usage in the process of intercultural communication, the awareness of the peculiarities of the English-language pharmaceutical discourse and vocabulary, as well as the understanding of the psychological prerequisites of the interconnected formation of lexical competence in reading and speaking.

English lexical competence in reading and speaking of future pharmacists is defined by their ability to adequately understand (when reading) and reproduce (linguistically) various lexical units (including terminological ones) in order to achieve personally and professionally significant communicative goals. The proposed method provides due consideration of the interests and experience of students; integrated formation of lexical skills of reading and speaking, as well as listening and writing; integration with professional subjects; training in metacognitive, cognitive, and compensatory strategies; increasing students' independence through the purposeful formation of their reflective abilities; development of professionally oriented English lexical competence in reading and speaking in three stages (introduction, during which students get acquainted with lexical units in and out of context, as well as develop their lexical awareness; training that promotes automated work of students with new vocabulary; application in the process of reading and speaking); using the proposed system of exercises, which consists of appropriate groups (ratio of stages) and subgroups. The proposed technique should improve the accuracy of understanding, the correctness of guessing the meaning of a lexical unit, the adequacy of the use of a lexical unit, and lexical concentration.

The objective of the paper was to analyze the effectiveness of a methodology aimed at developing professionally English-oriented lexical competence among future pharmacists as part of individual training.

## MATERIAL AND METHODS

This study was carried out in 2017–2018 among 57 students-prospective pharmacists at Bukovinian State Medical University. Participants were divided into two groups: experimental group 1 (EG1), with strict management of students' independent work, and experimental group 2 (EG2), with relatively strict management of students' independent work. 29 students were enrolled in EG1 and 28 in EG2. The effectiveness of the proposed methodology was assessed using the multifunctional statistical criterion ∏* – Fisher's angular transformation and the Mann-Whitney criterion.

Our methodology included: (1) students' interests and experience and securing the opportunity to select an individual mode of learning [6]; (2) developing reading, speaking, listening, and writing lexical skills; (3) interdisciplinary professional integration to determine English lexical competence content [7]; (4) stipulating students' autonomy through purposeful development of their intuitive skills [8]; (5) providing the system of control (including an indirect one) and self-control over the results of students' self-study [9].

We chose the working out learning model that assumed the application of the suggested methods during unguided work while learning English (Terms 1-3) throughout four stages: organizational (implemented at the beginning of Term 1), academic, assessment (at the end of each Term), and final (at the end of Term 3).

The criteria for assessing the level of English lexical competence in reading and speaking were: (1) accuracy of lexical units; (2) understanding and guess correctness (to determine the level of lexical competence in reading); (3) lexical variety and correctness of lexical units' usage (to check the level of lexical competence in speaking).

We used Fisher's angular transformation and the Mann-Whitney U-test, both of which indicated an excellent efficiency of the methodology with a relatively tight mode of learning management. The paper also contains methodological recommendations for developing prospective pharmacists' English lexical competence in reading and speaking through self-directed learning.

We developed the methodology based on the analysis of several foreign and domestic sources that consider students' individual study [10–13].

In EG1, the student's individual study was rigid and severely planned, and the tasks were checked during the classes. In the EG2 group, students' individual study was relatively rigid since the students could check some of the tasks using the answer keys. The results of the individual study were not constantly tested directly (sometimes indirectly), and the students could decide whether they had to do any additional exercises.

## RESULTS

The distribution of points by criteria is presented in Table 1. The results of the pre-and post-experimental assessments in EG1 and EG 2 are presented in Table 2. The learning rate was considered satisfactory for results greater than 0.7 points. However, the results of the pre-experimental assessment in EG1 and EG2 were 0.46 and 0.44, respectively, which proves that the students in both groups did not achieve the minimum learning rate.

**Table 1:** Criteria for assessing the level of prospective pharmacists' lexical competence in reading and speaking.

No.	Criteria	Points
**Lexical competence in reading**		
1.	Accuracy of understanding of lexical units	10
2.	Correctness of guessing the meaning of a lexical unit	10
**Lexical competence in speaking**		
3.	Correctness of using lexical units	20
4.	Lexical concentration	20
**Overall**		60

**Table 2:** Indicators of the pre-experimental and post-experimental assessment in EG1 and EG2.

Group	Criteria				Total points	Average rate
	Accuracy of understanding	Accuracy of understanding	Correctness of using	Correctness of using		
**Pre-experimental assessment**						
EG1	5.14	5.17	8.52	8.76	27.59	0.46
EG2	4.57	4.07	8.89	9.12	26.64	0.44
Maximum indicators	10	10	20	20	60	1
**Post-experimental assessment**						
EG1	8.21	8.24	14.62	14.35	45.41	0.76
EG2	8.68	8.57	16.07	16.46	49.79	0.83
Maximum indicators	10	10	20	20	60	1
**Increment**						
EG1	3.07	3.07	5.9	5.59	17.82	0.3
EG2	4.11	4.5	7.18	7.34	23.15	0.39

After the pre-experimental assessment, the equality of the experimental groups was tested using the multifunctional statistic criterion ∏* of Fisher's angular transformation [14]. The indicators of the pre-experimental and post-experimental assessment in EG1 and EG2 are presented in Table 2.

Two hypotheses were articulated:

H_0_: The percent of students in EG1 who achieved the learning rate according to the results of the pre-experimental assessment does not exceed that of EG2.

H_1_: The percent of students in EG1 who achieved the learning rate according to the results of the pre-experimental assessment is higher than that of EG2.

Table 3 compares the pre-experimental assessment results in EG1 and EG2.

**Table 3:** Comparison of the pre-experimental assessment results in EG1 and EG2.

Group index	With effect			Without effect			Overall number of students
	Number of students	Percent	φ	Number of students	Percent	φ	
EG1	2	6.9%	0.532	27	93.1%	2.610	29
EG2	3	10.7%	0.665	25	89.3%	2.475	28
Overall number of students	5	-	-	52	-	-	57

Let us calculate φ* _emp._ by the formula:


$$\varphi^{*}=(\varphi_{1}-\varphi_{2})\cdot \sqrt{\frac{n_{1}\cdot n_{2}}{n_{1}+n_{2}}}$$

where: ∏_1_ is the angle that corresponds to the higher percent (in our table, it is 10.7% of the EG2 students “with effect”); ∏_2_ is the angle that corresponds to the lower percent (6.9% of the EG1 students “with effect”); n_1_ is the number of students in EG1 (29); n_2_ is the number of students in EG2 (28).


$$\varphi_{emp.}=\left(0.665-0.532\right)\times \surd 812/57=0.506$$

**Figure 1:**
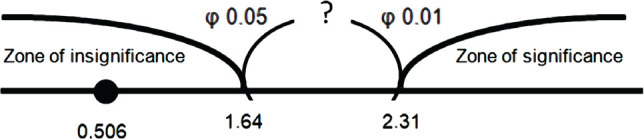
The axis of significance.

Furthermore, we made the axis of significance (Figure 1).

The value of Fisher's angular transformation was in the zone of insignificance (φ _emp._=0.506). Consequently, we rejected the hypothesis H1 and concluded that the percentage of students in EG1 who achieved the learning rate after the pre-experimental assessment did not exceed that of students in EG2. Hence, EG1 and EG2 groups were equal, and we could apply experimental learning in both, using the two suggested methods (with strict and relatively strict models of managing individual study).

In addition, the survey of the pre-experimental assessment showed students' poor performance rates in all criteria. Therefore, experimental learning requires equal attention to developing lexical competence in reading and speaking.

First, we compared the scores in EG1 and EG2 before and after the experiment. Both the strict model of managing individual study in EG1 was effective (φ _emp._=7.037) and the relatively strict model in φ _emp._=7.244 (Figure 1).

Finally, we had to determine whether Option B was more effective than Option A, which was indicated by the outcomes of the post-experimental assessment presented in Table 2.

Two hypotheses were again articulated:

H_0_: The percentage of students in EG2 who achieved the learning rate does not exceed that of EG1, according to the results of the post-experimental assessment.

H_1_: The percentage of students in EG2 who achieved the learning rate was higher than that of EG1, according to the results of the post-experimental assessment.

Since most EG2 and a great number of EG1 students had a learning rate score of 0.8 and more, we considered it effective.

Table 3 compares the post-experimental assessment results in EG1 and EG2.

**Figure 2:**
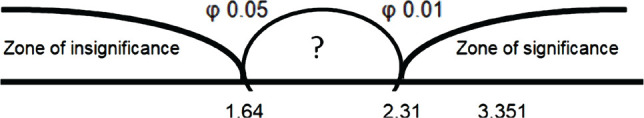
The axis of significance.

The obtained φ* _emp._=3.725 belongs to the zone of significance (Figure 2).

So, hypothesis H1 is accepted, in compliance with the percent of EG2 students who achieved the post-experimental assessment learning rates, which was higher than that of EG1. Accordingly, a relatively strict model of managing students' individual study had greater effectiveness.

EG2 students had a higher learning rate increment than EG1. It is also worth pointing out that the above increment is relatively balanced for various criteria. The EG2 average learning rate by criterion 1 (accuracy of understanding) equaled 0.46 before the experiment and 0.87 – after it (in EG1 - 0.51 and 0.82, respectively). The average learning rate in EG2 by criterion 2 (correctness of guessing the meaning of a lexical unit) was 0.41 before and 0.86 – after the experiment (in EG1 - 0.52 and 0.82, respectively). The average learning rate in EG2 by criterion 3 (correctness of using) - 0.44 and 0.8 before and after the experiment (in EG1 - 0.43 and 0.73). Eventually, criterion 4 (lexical concentration) was marked with the following indicators: EG2 - 0.46 and 0.82 (EG1 – 0.44 and 0.72). These outcomes emphasize the efficacy of the suggested system of exercises. On the other hand, EG1 participants had better scores regarding criteria 1 and 2, which tested students' competence in reading. Given the strict model of managing students' individual study in EG1, we might assume that teacher's permanent control over the process ensured students' clear comprehension of the learned lexical units, as well as the correctness of guessing their meanings. The learning rate of lexical skills in speaking was considerably better in EG2 students than in EG1. This phenomenon might be explained by EG2 students' having more opportunities for creative approach, which is essential for the productive types of speech activities.

## DISCUSSION

Ukrainian scholars have carried out numerous investigations on different aspects of developing English lexical competence in various fields: prospective teachers [15, 16], economists [17, 18], finance [19], engineers [20], and doctors [21–23].

Today, there is an urgent need to identify scientific methods for forming pharmacists' professionally oriented English lexical competence, especially due to a limited number of assigned classroom hours.

The conducted experiment consisted of the following stages: (1) forming the experimental groups; (2) determining the equality of EG1 and EG2 via the pre-experimental assessment; (3) experimental learning; (4) conducting the post-experimental assessment; (5) statistical processing of the obtained results; (6) analysis and interpretation of results; (7) substantiating the criteria for assessing the level of lexical competence in reading and learning.

Regarding the correctness of using lexical units, the participants received points in reference to the number of mistakes they made in using or pronouncing a lexical unit. 1 mistake allowed the students to get 10 points, 2 mistakes – 9 points, 3 – 8, 4 – 7, 5 – 6, 6 – 5, 7 – 4, 8 – 3, 9 – 2, 10 – 1.

According to the criterion of lexical concentration, for each correctly new lexical unit used, students received 1 point. Comparing the research outcomes with those of other scholars (who selected either the level of students' autonomy or the mode of managing students' individual study as the variable value), we might draw the following conclusions. It is more convenient to apply a rigid model of managing students' individual study during the first year of learning, whereas a relatively rigid mode is preferable for second-year students [24]. Investigating the development of prospective lawyers' German lexical competence during self-study using information technologies, there was a greater efficiency with methods that involve the maximum application of computer programs [25]. However, it is assumed that the obtained results are stipulated, firstly, by the specifics of using a computer program (the latter presupposes regulating students' actions) and, secondly, by its orientation on receptive lexical competence.

Our study does not contradict the results of other scientific investigations. Moreover, our results highlight the necessity for developing students' learning autonomy and the expediency of providing them with possibilities for creative work, as it is preferable to avoid a rigid model of managing students' individual study when developing their lexical competence in speaking.

## CONCLUSION

Our data demonstrate the effectiveness of the suggested methodology for forming pharmacists' English lexical competencies in reading and speaking, as well as the expediency of applying a relatively rigid mode of managing students' individual study. The potential significance of further investigations in this field might lie in elaborating methods for improving prospective pharmacists' English lexical competence in listening and writing.
